# The relationship between academic resilience and depression among Chinese adolescents: a chain mediation model of perceived academic pressure and anxiety

**DOI:** 10.3389/fpubh.2026.1813364

**Published:** 2026-08-20

**Authors:** Guangqiang Wang, Huayang Zhang, Yuxue Hou, Jia Wei

**Affiliations:** 1 Department of Educational Management, Faculty of Education, East China Normal University, Shanghai, China; 2 School of Digital Media, Shenzhen Polytechnic University, Shenzhen, China; 3 Teachers' College, Beijing Union University, Beijing, China

**Keywords:** academic resilience, anxiety, Chinese adolescents, depression, perceived academic pressure

## Abstract

The relationship between academic resilience and adolescents' depression remains unanalyzed. This study aims to examine the relationship between academic resilience and adolescent depression in China based on the conservation of resources theory, revealing the mediating roles of perceived academic pressure and anxiety. This study employs data from the China Education Panel Survey (CEPS; 2014–2015), which includes 7,328 Chinese adolescents. Model 6 of the PROCESS macro program in SPSS 24.0 software and the bias-corrected percentile bootstrap method were employed to examine the mediating effect of perceived academic pressure and anxiety. The results showed that academic resilience was significantly and negatively related to depression, perceived academic pressure, and anxiety among adolescents. Adolescents' depression was significantly and positively related to both perceived academic pressure and anxiety. Perceived academic pressure and anxiety not only served as separate mediators between academic resilience and adolescents' depression but also played a chain-mediating role. In theory, this study revealed the mediating roles of perceived academic pressure and anxiety, thereby enriching the existing literature. In practice, schools should enhance students' academic resilience and reduce their perceived academic pressure and anxiety, thereby effectively alleviating their depression.

## 1 Introduction

Adolescent depression has received widespread attention globally (1–3), and China is no exception (4). According to the *Report on National Mental Health Development in China (2019–2020)*, the detection rate of depression among Chinese adolescents is 24.6%, with mild depression accounting for 17.2% and severe depression for 7.4% (5). A recent meta-analysis indicated that the overall detection rate of depression among Chinese middle school students is 24% (6). Depression, as a common mental disorder, refers to an individual's negative reaction to adverse situations and events (7), manifesting primarily as somatic symptoms such as persistent sadness, self-criticism, feelings of meaninglessness, lethargy, and poor sleep quality (8). Depression can seriously affect the healthy development of adolescents, leading not only to a decline in academic performance (9) but also to school dropout (10), problematic internet use (11), problematic social media use (12), substance use (13), and even suicidal and self-harming behaviors (14). Therefore, identifying predictors of adolescent depression and developing effective programs and strategies to alleviate adolescent depression represents an urgent issue that scholars must address.

Previous studies have primarily examined the critical role of psychological resilience in alleviating depression (15, 16), neglecting the impact of academic resilience on adolescent depression. Academic resilience refers to a student's ability to successfully cope with setbacks in the learning process (17). High academic resilience contributes to adolescents' ability to overcome academic-related stress and challenges (18) while increasing their engagement and achievement (19, 20), thereby effectively reducing depression. Thus, academic resilience may be a significant factor influencing adolescent depression. Furthermore, the mechanisms and boundary conditions by which academic resilience influences adolescent depression remain unclear. According to the conservation of resources theory (COR) (21, 22), academic resilience, as a crucial personal resource (17), enhances adolescents' resource accumulation, mitigates resource depletion, and reduces their stress responses (e.g., academic stress and anxiety), thereby alleviating depression. Additionally, prior research indicates that academic pressure and anxiety serve as key triggers for depression (3, 23). Thus, academic pressure and anxiety may mediate the relationship between academic resilience and adolescent depression.

In conclusion, previous studies have rarely explored how academic resilience affects adolescent depression. This study aims to investigate the relationship between academic resilience and depression among Chinese adolescents based on COR theory, revealing the mediating roles of perceived academic pressure and anxiety between them. This study offers significant theoretical implications. First, by empirically examining the relationship between academic resilience and adolescent depression, it introduces novel insights for alleviating adolescent depression and fills the gaps in related literature. Second, the mechanisms by which academic resilience affects adolescent depression remain inadequately explored. The present study further reveals the mediating roles of perceived academic pressure and anxiety, offering a comprehensive analytical framework to enhance the understanding of how academic resilience influences adolescent depression.

## 2 Literature review and hypotheses

### 2.1 Academic resilience and depression

Resilience refers to the ability of an individual to adapt positively and achieve their goals in the face of stress and adversity (24, 25). Academic resilience is commonly defined as a student's ability to effectively cope with challenges, setbacks, adversity, and stress during the learning process (26). According to the COR, individuals are inclined to protect and acquire resources (21, 22). Academic resilience serves as an essential personal resource that helps adolescents avoid the threat of resource loss and cope with actual losses (17). Specifically, academic resilience significantly enhances adolescents' learning engagement (17), academic achievement (27), and life satisfaction (28). It also allows students to experience greater academic meaning (29) and wellbeing (30) while mitigating academic burnout (31). These positive learning experiences and states effectively prevent depression in adolescents. Furthermore, academic resilience improves adolescents' ability to adapt to changes and challenges in the learning environment (32), enabling them to cope more effectively with difficulties and setbacks (26), thereby reducing the negative emotions that arise (31). Numerous studies have supported the notion that academic resilience substantially alleviates adolescent depression. For instance, Anderson et al. (1) demonstrated that resilience not only reduced adolescent depression but also mediated the relationship between bullying and depression. Similarly, Gong et al. (33) found that resilience was significantly negatively correlated with depression in Chinese adolescents, mediating and moderating the relationship between personality traits and depression. Therefore, based on the above analysis, the following hypothesis is proposed:

*H1*: Academic resilience is significantly and negatively related to adolescents' depression.

### 2.2 The mediating effect of perceived academic pressure

Academic pressure refers to the psychological tension that arises when students face various demands and expectations during the learning process. This stress may stem from academic demands, test loads, and excessive homework (3). From the perspective of COR (21, 22), academic resilience, as a psychological resource (17), can effectively alleviate adolescents' stress response, that is, academic pressure. Adolescents with high resilience are typically confident in their problem-solving abilities (34). Even in adverse or unfavorable academic situations, they can remain calm and motivated, adopting strategies to cope effectively with setbacks and stress (31). Existing literature demonstrated that academic resilience can indirectly affect undergraduate students' academic performance by reducing academic stress (18). Studies by Berdida (35), Okechukwu et al. (36), Ho et al. (37), and Versteeg and Kappe (38) consistently showed that resilience was significantly and negatively related to students' academic stress. Meanwhile, numerous studies indicated that academic pressure was a critical factor contributing to depression in adolescents (37). For example, Fu et al. (39) found that adolescents' perceived academic stress was significantly and positively correlated with their depression, and this relationship was mediated by parent-child communication and interaction. Similarly, Jiang et al. (3) suggested that academic stress significantly and positively influenced Chinese adolescents' depression. Therefore, based on the above analysis, the following hypotheses are proposed:

*H2a:* Academic resilience is significantly and negatively related to adolescents' perceived academic pressure
*H2b:* Perceived academic pressure is significantly and positively related to adolescents' depression
*H2c:* Perceived academic pressure mediates the relationship between academic resilience and adolescents' depression.

### 2.3 The mediating effect of anxiety

Anxiety refers to an individual's subjective experience of fear and worry, characterized by unpleasant feelings of inner turmoil (40). According to the COR theory, anxiety represents a stress response triggered by perceived resource threats among adolescents, resulting in negative outcomes such as academic burnout (41), reduced academic engagement (42), and lower academic achievement (43). Meanwhile, academic resilience, as a personal characteristic resource, helps mitigate adolescents' resource depletion and alleviates their stress response, particularly anxiety. Fathalla (44) demonstrated a significant negative relationship between academic resilience and test anxiety. Hayat et al. (45) found that academic resilience negatively impacted medical students' test anxiety. Moreover, other studies also showed that resilience was negatively related to adolescent anxiety (46–48). Meanwhile, anxiety is widely acknowledged as an important antecedent variable of depression (48–50). The literature indicated that higher levels of anxiety in adolescents were significantly associated with an elevated likelihood of developing depression (51). For instance, Wang et al. (52) found that anxiety was positively correlated with adolescent depression. Hill et al. (53) demonstrated that adolescent anxiety not only positively affected depression but also mediated the relationship between depression and suicidal ideation. Therefore, based on the above analysis, we believe that anxiety mediates the relationship between academic resilience and adolescent depression. Therefore, based on the above analysis, the following hypotheses are proposed:

*H3a:* Academic resilience is significantly and negatively related to adolescents' anxiety
*H3b*: Anxiety is significantly and positively related to adolescents' depression
*H3c*: Anxiety mediates the relationship between academic resilience and adolescents' depression.

### 2.4 The chain-mediating effect of perceived academic pressure and anxiety

In fact, a strong correlation exists between academic pressure and adolescent anxiety. Adolescents possess limited psychological resources for learning, which may be rapidly depleted when they encounter academic stress and challenges. Without effective stress relief or timely access to and preservation of resources, psychological burdens can intensify, ultimately triggering anxiety. Many studies indicated that academic pressure was a significant positive predictor of anxiety (54), suggesting that individuals experiencing high levels of academic pressure were more likely to develop anxiety (55). Chen et al. (56) found that academic stress was significantly and positively correlated with both anxiety and depression among Chinese middle school students. Similarly, Li et al. (57) demonstrated that adolescents' anxiety was not only positively associated with academic stress but also mediated the relationship between academic stress and aggression. Based on these analyses, it can be inferred that academic stress and anxiety play a chain-mediating role in the relationship between academic resilience and adolescent depression. Therefore, based on the above analysis, the following hypotheses are proposed:

*H4a*: Perceived academic pressure is significantly and positively related to adolescents' anxiety
*H4b*: Perceived academic pressure and anxiety have a chain-mediating effect in the relationship between academic resilience and adolescents' depression.

**Figure 1:**
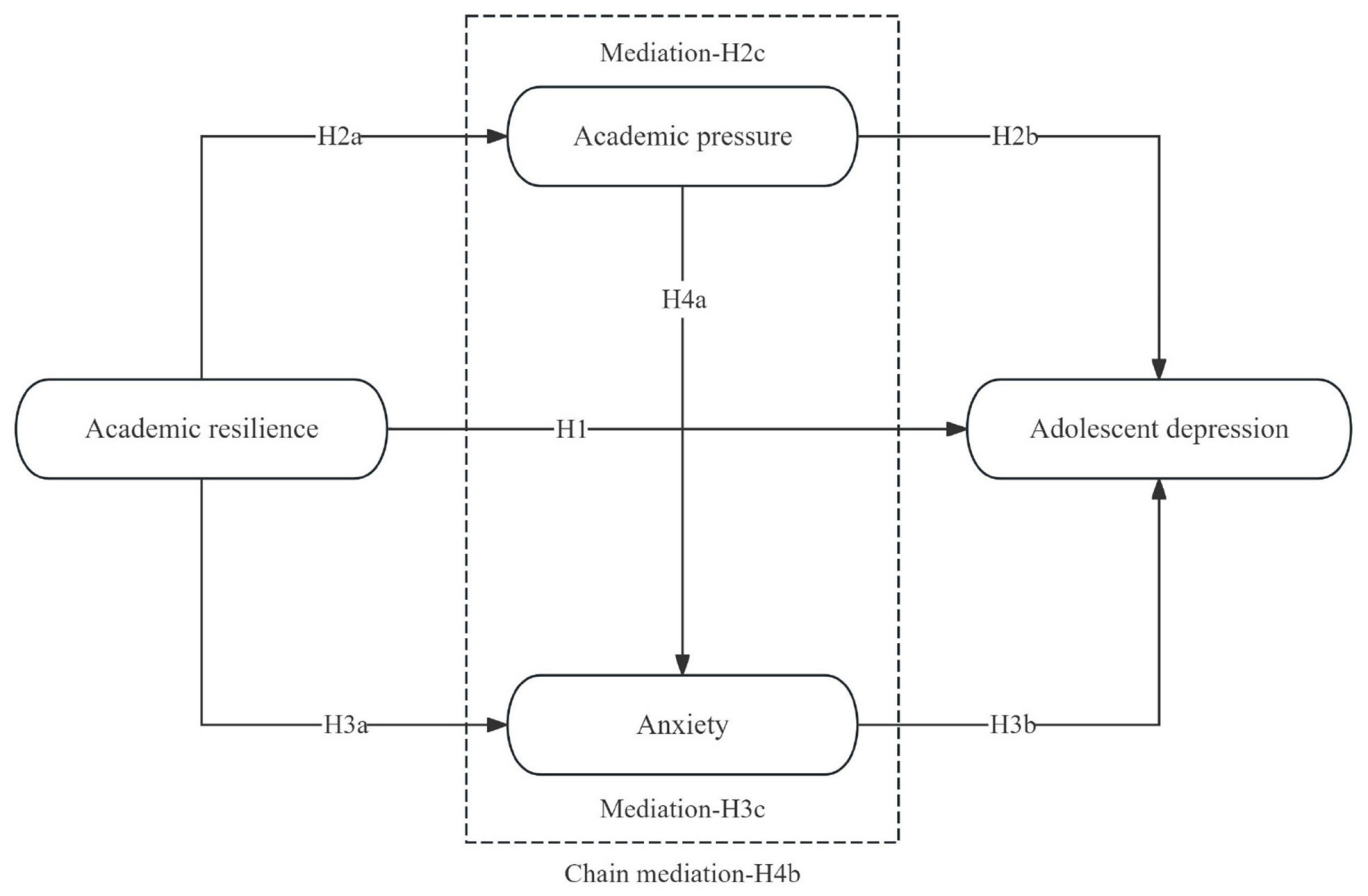
Research model.

Based on the above research hypotheses, a research model is presented in Figure 1.

## 3 Methods

### 3.1 Participant

This study utilized tracking data from the 2014–2015 academic year of the China Education Panel Survey (CEPS), a large-scale nationally representative tracking survey designed to reveal the impact of macro- and micro-social structures on individual educational outcomes. The program focused on questionnaires administered to students in the 1st year of grades 7 and 9. The baseline survey for CEPS was conducted during the 2013–2014 academic year, while the tracking survey was conducted during the 2014–2015 academic year. The sampling procedure involved randomly selecting 28 county-level units nationwide and subsequently selecting four schools within each county that offered grades 7 and/or 9, resulting in a total of 112 schools. In each school, four classes were randomly selected, comprising two seventh-grade classes and two ninth-grade classes, with all students in the selected classes included in the sample.

Although the data were collected several years ago, this dataset remains highly relevant for research on adolescent mental health. First, from a contextual perspective, the structural characteristics of China's education system—marked by high-stakes exams and intense academic competition—have remained largely stable. Therefore, the significant academic pressure, anxiety, and depressive symptoms experienced by Chinese adolescents remain a pressing issue. Second, from an empirical perspective, many recent studies have utilized this database (58, 59), and research by Bai et al. (60) demonstrates the ongoing applicability and reliability of the CEPS dataset in studies of adolescent depression. Consequently, using the CEPS to elucidate how academic resilience mitigates academic pressure, anxiety, and depression remains of profound significance for educational interventions. In this study, the valid sample consisted of 7,328 students, of whom 3,729 were male (50.89%) and 3,599 were female (49.11%). The survey revealed that 3,352 students (45.74%) were only children, while 3,976 (54.26%) were not. Additionally, 6,617 students (90.30%) reported that their parents had a good relationship, whereas 711 students (9.70%) reported a poor parental relationship. Regarding family financial conditions, 1,027 students (14.01%) came from families experiencing very difficult or relatively difficult financial circumstances, 5,402 students (73.72%) came from families with average financial conditions, and 899 students (12.27%) came from relatively wealthy or very wealthy backgrounds.

### 3.2 Measures

#### 3.2.1 Academic resilience

Measures of academic resilience focus on students' responses to emotional problems, academic stress, and other setbacks in school (26). Following Fang et al. (19), academic resilience in this study was assessed using three items, such as “I would try my best to finish even the homework I dislike.” A four-point Likert scale was employed, ranging from “1 = completely disagree” to “4 = completely agree,” with higher scores reflecting greater levels of academic resilience among students. The Cronbach's alpha coefficient for the scale was 0.81, indicating acceptable reliability.

#### 3.2.2 Perceived academic pressure

According to Wang and Jiang (61), this study also utilized the question, “Do you currently find it difficult to learn mathematics, Chinese, and English?” to assess perceived academic pressure. A four-point Likert scale was employed to rate the items, ranging from “1 = especially difficult” to “4 = not at all difficult.” The items were reverse scored, such that higher scores reflected greater levels of academic pressure.

#### 3.2.3 Anxiety

Referring to Li et al. (62) and Zhang et al. (63), anxiety in this study was measured using three items: feeling nervous, being overly worried, and anticipating that something bad might happen within seven days. A five-point Likert scale was employed, ranging from “1 = never” to “5 = always,” with higher scores indicating greater levels of anxiety among students. The Cronbach's alpha coefficient for the scale was 0.81, indicating good reliability.

#### 3.2.4 Depression

According to the study of Wang and Jiang (61), this study also assessed depression using six items: feeling down, being too depressed to concentrate, feeling unhappy, perceiving life as meaningless, lacking the energy to complete tasks, and experiencing sadness. A five-point Likert scale was employed, ranging from “1 = never” to “5 = always,” with higher scores indicating greater levels of depression. The Cronbach's alpha coefficient for the scale was 0.92, indicating excellent reliability.

#### 3.2.5 Control variables

Drawing from previous studies, the present study included gender, only-child status, parental relationship, and family economic conditions as control variables in the research model to enhance understanding of how academic resilience influences adolescents' depression.

### 3.3 Data analysis

SPSS 24.0 software was used to analyze the data. First, a common method bias test, descriptive statistics, and correlation analyses were performed using SPSS 24.0. Second, Model 6 in the PROCESS macro program of SPSS 24.0 was used to examine the relationships among academic resilience, perceived academic pressure, anxiety, and adolescents' depression. Finally, the significance of the mediating effects of perceived academic pressure and anxiety was also tested using the bias-corrected percentile bootstrap method. Their mediating effects were established when the 95% confidence interval (CI) did not contain zero. A *p*-value of <0.05 (two-tailed) was considered statistically significant.

## 4 Results

### 4.1 Common method bias tests

In addition to procedural controls, a common method bias test was also conducted using the Harman single-factor method. The results of the unrotated exploratory factor analysis indicated that four factors with eigenvalues greater than 1 were extracted, and the maximum explained variance of these factors was 27.806%, well below the threshold of 40%. Therefore, common method bias was not a significant concern in this study.

### 4.2 Descriptive statistics and correlation analysis

Table 1 shows that adolescents' academic resilience was significantly and negatively correlated with depression, perceived academic pressure, and anxiety (*r* = −0.161, *p* < 0.001; *r* = −0.243, *p* < 0.001; *r* = −0.082, *p* < 0.001). Depression was significantly and positively related to perceived academic pressure and anxiety (*r* = 0.253, *p* < 0.001; *r* = 0.660, *p* < 0.001). Perceived academic pressure was also significantly and positively associated with anxiety (*r* = 0.177, *p* < 0.001).

**Table 1:** Descriptive statistics and correlation analysis (*N* = 7,328).

Variable	*M*	SD	1	2	3	4
1=Academic resilience	3.895	0.992	1			
2=Depression	2.156	0.899	−0.161^***^	1		
3=Perceived academic pressure	2.891	0.859	−0.243^***^	0.253^***^	1	
4=Anxiety	2.240	0.943	−0.082^***^	0.660^***^	0.177^***^	1

^***^p < 0.001 (two-tailed); M, mean; SD, standard deviation.

### 4.3 The chain mediation effect of perceived academic pressure and anxiety

Model 6 of the PROCESS macro program in SPSS was utilized to test the chain mediating role of perceived academic pressure and anxiety between academic resilience and depression. According to Table 2, academic resilience was significantly and negatively related to depression, perceived academic pressure, and anxiety (β = −0.128, *p* < 0.001; β = −0.189, *p* < 0.001; β = −0.034, *p* < 0.001). Perceived academic pressure was significantly and positively correlated with depression and anxiety (β = 0.107, *p* < 0.001; β = 0.174, *p* < 0.001). Anxiety was significantly and positively associated with depression (β = 0.597, *p* < 0.001). Therefore, hypotheses H1, H2a, H2b, H3a, H3b, and H4a were supported.

**Table 2:** Test of the mediating effect of perceived academic pressure and anxiety.

Outcome variables	Independent variables	*R*	*R* ^2^	*F*	β	SE	*t*	*P*
Depression		0.242	0.059	90.938^***^				
	Gender				−0.005	0.020	−0.231	0.818
	Only child status				0.100	0.021	4.762^***^	0.000
	Parental relationship				0.397	0.035	11.438^***^	0.000
	Family financial conditions				−0.140	0.018	−7.992^***^	0.000
	Academic resilience				−0.128	0.010	−12.351^***^	0.000
Perceived academic pressure		0.367	0.135	228.358^***^				
	Gender				−0.017	0.019	−0.886	0.376
	Only child status				0.245	0.019	12.787^***^	0.000
	Parental relationship				0.141	0.032	4.429^***^	0.000
	Family financial conditions				−0.289	0.016	−18.090^***^	0.000
	Academic resilience				−0.189	0.010	−19.935^***^	0.000
Anxiety		0.201	0.041	51.595^***^				
	Gender				0.002	0.022	0.072	0.943
	Only child status				−0.015	0.022	−0.682	0.495
	Parental relationship				0.273	0.037	7.422^***^	0.000
	Family financial conditions				−0.024	0.019	−1.285	0.199
	Academic resilience				−0.034	0.011	−3.024^**^	0.003
	Perceived academic pressure				0.174	0.014	12.869^***^	0.000
Depression		0.685	0.469	923.816^***^				
	Gender				−0.002	0.015	−0.140	0.889
	Only child status				0.057	0.016	3.603^***^	0.000
	Parental relationship				0.205	0.026	7.803^***^	0.000
	Family financial conditions				−0.064	0.013	−4.794^***^	0.000
	Academic resilience				−0.068	0.008	−8.490^***^	0.000
	Perceived academic pressure				0.107	0.010	10.986^***^	0.000
	Anxiety				0.597	0.008	71.969^***^	0.000

^**^p < 0.01, ^***^p < 0.001 (two-tailed).

**Figure 2:**
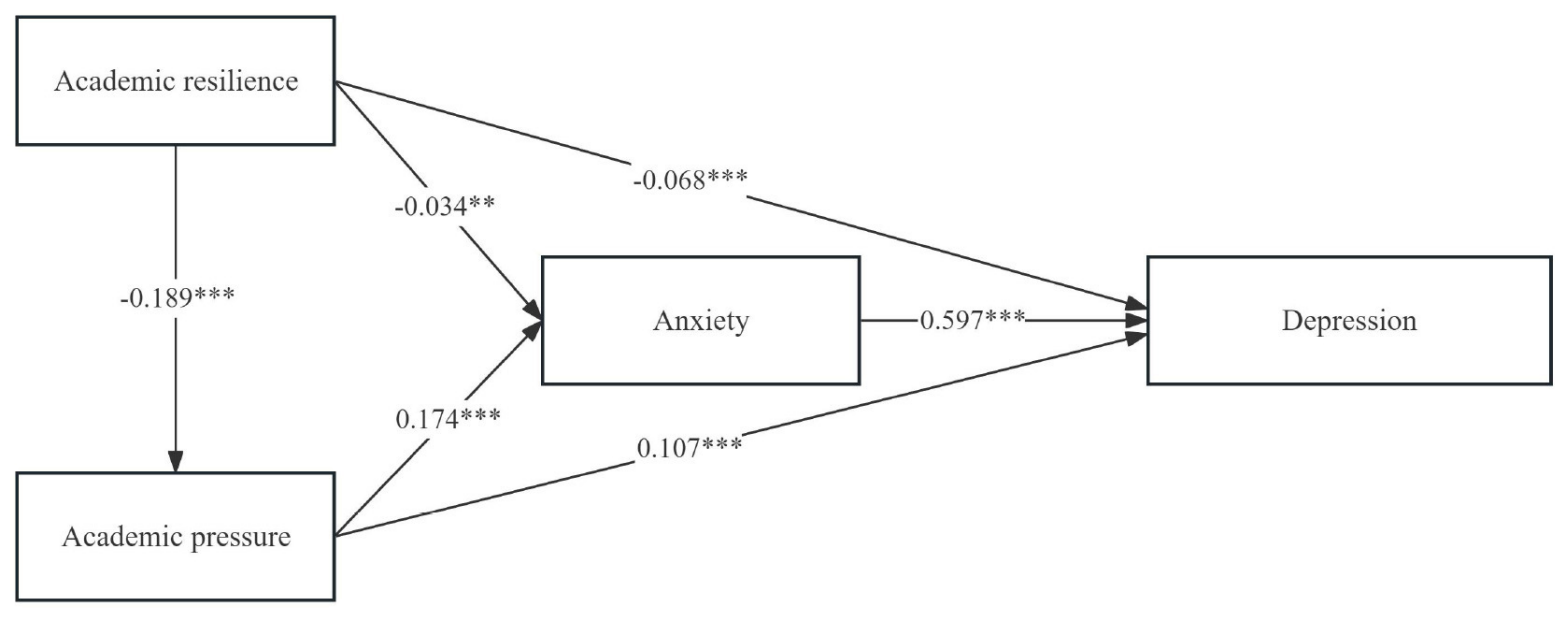
The effects of academic resilience on depression. **p < 0.01, ***p < 0.001.

The chain mediation effect of perceived academic pressure and anxiety was further examined using the bias-corrected bootstrap method with 5,000 sample replications. Table 3 indicates that the value of the direct effect was −0.068 with a 95% CI excluding 0, indicating a significant direct effect of academic resilience on depression. The mediating effect of perceived academic pressure was −0.020 with a 95% CI excluding 0, indicating that perceived academic pressure partially mediated the relationship between academic resilience and depression. The mediating effect of anxiety was −0.020 with a 95% CI excluding 0, indicating that anxiety partially mediated this relationship as well. The mediating effect of perceived academic pressure and anxiety combined was −0.020 with a 95% CI excluding 0, indicating a chain mediation effect of perceived academic pressure and anxiety between academic resilience and depression. Therefore, hypotheses H2c, H3c, and H4b were supported. The specific pathways through which academic resilience affects depression are illustrated in Figure 2.

**Table 3:** Total, direct, and mediation effects of perceived academic pressure and anxiety.

Path	Effect	SE	Bias-corrected 95%CI		Ratio
			Lower	Upper	
Total effect	−0.128	0.011	−0.149	−0.105	
Direct effect	−0.068	0.009	−0.085	−0.051	53.13%
Total indirect effect	−0.060	0.008	−0.075	−0.045	46.88%
Indirect 1	−0.020	0.002	−0.025	−0.016	15.63%
Indirect 2	−0.020	0.007	−0.035	−0.006	15.63%
Indirect 3	−0.020	0.002	−0.024	−0.016	15.63%

Indirect 1: Academic resilience → perceived academic pressure → depression; Indirect 2: Academic resilience → anxiety → depression; Indirect 3: Academic resilience → perceived academic pressure → anxiety → depression.

## 5 Discussion

Depression is a common and serious mental health issue among adolescents (61). Previous studies have shown that resilience effectively alleviates adolescent depression (1). However, the specific association between academic resilience and adolescent depression has not received much attention. Therefore, based on COR theory, this study revealed the mediating roles of perceived academic pressure and anxiety between academic resilience and depression among Chinese adolescents, contributing significantly to the existing literature.

First, the results showed that academic resilience was significantly and negatively related to adolescents' depression. This implies that higher academic resilience is associated with a lower likelihood of adolescents experiencing depression. This finding aligns with the interpretation of COR theory. Academic resilience refers to an individual's ability to adapt positively and complete learning tasks despite adversity (26), functioning as an important personal resource in adolescents' learning processes (17). According to COR theory, the acquisition and preservation of resources help individuals avoid resource loss while enabling further investment in resources (21, 22). Academic resilience not only mitigates negative emotions resulting from resource depletion but also enhances adolescents' mental health. Furthermore, this result is consistent with findings from similar studies (1, 64). Notably, this finding is not limited to adolescents in China but has been widely validated in other countries and regions. For instance, Ho et al. (37) found that resilience was a significant negative predictor of depression in a study involving 1,336 Vietnamese adolescents. Kokou-Kpolou et al. (65) noted that resilience served as a buffer and a moderator in the relationship between perceived stress and depression among French university students. Additionally, the study of Versteeg and Kappe (38) indicated a significant negative association between resilience and depression among students in the Netherlands. These findings suggest that resilience is a protective factor for mental health independent of cultural context. This provides theoretical support for cross-cultural research on academic resilience and highlights the need for further exploration of its positive effects in diverse cultural settings.

Second, the results indicated that perceived academic pressure mediated the relationship between academic resilience and depression among Chinese adolescents. This implies that academic resilience can indirectly alleviate adolescent depression by reducing academic stress. First, academic resilience was significantly negatively correlated with perceived academic pressure. This suggests that as adolescents become more confident in their ability to recover quickly and adapt positively in the face of high levels of stress, they experience less academic stress. This result aligns with findings from similar studies (66). However, other research presents a different perspective. For instance, Ho et al. (37) and Berdida and Grande (67) found that academic stress may significantly diminish resilience. Similarly, Zheng et al. (68) demonstrated that while resilience significantly reduced daily stress, daily stress also negatively impacted resilience. These findings imply a potential reciprocal causal relationship between academic resilience and academic pressure that warrants further exploration in future studies. Second, perceived academic pressure was significantly and positively associated with depression in adolescents. Higher levels of academic pressure in adolescents were linked to a greater likelihood of depression. This finding is consistent with the studies of Jiang et al. (3) and Fu et al. (39). Thus, academic pressure represents a key factor through which academic resilience alleviates depression in adolescents.

Third, the results demonstrated that anxiety served as a mediator in the relationship between academic resilience and depression among Chinese adolescents. This finding implies that academic resilience indirectly influences adolescent depression through the reduction of anxiety. First, academic resilience was significantly negatively correlated with anxiety, consistent with previous research (45, 69). Adolescents with high academic resilience demonstrate greater competence and ease in addressing academic challenges while experiencing reduced fear and worry compared to their less resilient peers. Moreover, some studies suggested that anxiety exerts a negative influence on resilience. For instance, Qian and Yu (70) found that Chinese language learning anxiety among international students in China predicted mental health outcomes through the mediating effect of academic resilience. Similarly, Seçer and Ulaş (71) reported that academic resilience fully mediated the relationship between anxiety sensitivity and high school students' school attachment. Therefore, a bidirectional causal relationship may exist between academic resilience and anxiety. Second, anxiety was significantly and positively associated with depression among Chinese adolescents, aligning with earlier research (72). Higher levels of anxiety corresponded to increased levels of depression (53). In conclusion, anxiety plays a crucial role in the mechanism through which academic resilience affects adolescent depression.

Finally, the results also showed that perceived academic pressure and anxiety exerted a chain mediating effect between academic resilience and depression among Chinese adolescents. This finding indicates that academic resilience, as a vital psychological resource, indirectly reduces adolescents' anxiety by initially alleviating their academic stress. When academic pressure is reduced, students experience less inner panic and worry in their studies, and this emotional relief can effectively prevent them from falling into a depressive state characterized by low mood and loss of interest in life (54). In addition, this study identified a significant positive correlation between perceived academic pressure and anxiety. This result is consistent with previous research, suggesting that greater academic pressure corresponds to heightened levels of anxiety (57). This relationship underscores the importance of alleviating academic pressure to reduce anxiety in adolescents.

## 6 Implications and limitations

This study has important practical implications. First, to enhance adolescents' academic resilience, schools may implement structured, multi-week growth mindset interventions—such as mindset workshops focused on reframing academic setbacks as milestones in a learning journey—combined with stress-reduction programs guided by cognitive behavioral therapy. These programs should explicitly instruct adolescents to view poor test scores or high-stakes stress as surmountable challenges. Additionally, schools should formally establish peer support groups and teacher-guided mechanisms centering on the sharing of emotional regulation and coping strategies, transforming social resources into practical and effective means of alleviating academic anxiety. Second, to alleviate academic pressure among adolescents, school administrators must reform curriculum assessment and workload management. Rather than excessively relying on high-stakes summative exams, they should adopt diversified formative assessment models that recognize students' incremental progress. This structural shift must be implemented through institutional policies, such as reducing homework time and increasing opportunities for rest and extracurricular activities. Finally, schools should conduct routine mental health screenings that target early signs of academic burnout, academic helplessness, or disengagement from learning. For students identified through these screenings, schools can provide tailored group interventions that focus on teaching practical stress management techniques, such as cognitive restructuring for test anxiety and progressive muscle relaxation.

This study has several limitations. First, the cross-sectional design restricted the ability to infer causal relationships among the variables. Future research should employ experimental or longitudinal designs to explore these relationships further. Second, this study primarily employed a single-level analytical framework to examine the relationships among these variables, which may not adequately account for potential clustering effects. Future research should utilize multilevel models to explore these relationships further. Third, drawing on relevant research, this study utilized selected items to measure academic resilience and perceived academic pressure among adolescents, which may not comprehensively reflect the multidimensional nature of academic resilience and academic pressure. Future research should employ established academic resilience and perceived academic pressure scales to conduct analyses and further validate the study's findings. Fourth, this study primarily controlled for gender, only child status, parental relationship, and family financial conditions in its analysis. However, age and grade level were not controlled. It is recommended that future studies include age and grade level as control variables for further discussion. Finally, this study focused on the relationship between academic resilience and adolescent depression within a Chinese context, which precluded cross-cultural comparisons. Future studies should validate these findings in other regional and cultural contexts to enhance their generalizability.

## 7 Conclusions

First, academic resilience was significantly and negatively correlated with adolescents' perceived academic pressure, anxiety, and depression. Second, perceived academic pressure mediated the relationship between academic resilience and adolescents' depression. Third, anxiety mediated the relationship between academic resilience and adolescents' depression. Finally, perceived academic pressure and anxiety played a chain-mediating role between academic resilience and adolescents' depression.

## Data Availability

The raw data supporting the conclusions of this article will be made available by the authors, without undue reservation.
